# Small Structural Differences Govern the Carbonic Anhydrase II Inhibition Activity of Cytotoxic Triterpene Acetazolamide Conjugates

**DOI:** 10.3390/molecules28031009

**Published:** 2023-01-19

**Authors:** Toni C. Denner, Niels Heise, Julian Zacharias, Oliver Kraft, Sophie Hoenke, René Csuk

**Affiliations:** Organic Chemistry, Martin-Luther University Halle-Wittenberg, Kurt-Mothes, Str. 2, D-06120 Halle (Saale), Germany

**Keywords:** triterpenoic acid, carbonic anhydrase II, acetazolamide conjugate, cytotoxicity

## Abstract

Acetylated triterpenoids betulin, oleanolic acid, ursolic acid, and glycyrrhetinic acid were converted into their succinyl-spacered acetazolamide conjugates. These conjugates were screened for their inhibitory activity onto carbonic anhydrase II and their cytotoxicity employing several human tumor cell lines and non-malignant fibroblasts. As a result, the best inhibitors were derived from betulin and glycyrrhetinic acid while those derived from ursolic or oleanolic acid were significantly weaker inhibitors but also of diminished cytotoxicity. A betulin-derived conjugate held a K_i_ = 0.129 μM and an EC_50_ = 8.5 μM for human A375 melanoma cells.

## 1. Introduction

**Figure 1:**
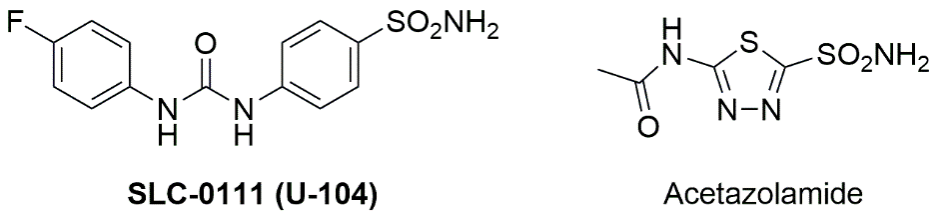
Structure of well-established CA inhibitors SLC-0111 and acetazolamide.

The ubiquitous metalloenzymes carbonic anhydrases (CAs) [1,2,3,4,5,6] are present in bacteria [7,8] and fungi [9,10,11,12], plants and animals. Inhibitors of these enzymes have been clinically exploited for decades, and the discovery of multiple human isoforms [13,14,15,16,17,18] has led to many new applications and the development of new therapeutic principles, among them antiglaucoma [19,20,21] and antitumor drugs but also antiepileptic [22,23,24,25,26] and antiobesity drugs [27,28,29] as well as agents for the management of Alzheimer’s disease [30,31], neuropathic pain, cerebral ischemia, and some forms of arthritis [32,33,34]. Furthermore, the development of inhibitors for bacterial carbonic anhydrases is thought as a new concept to develop antibacterial drugs [35,36,37,38,39,40,41,42]. In addition, drug conjugates were investigated for their ability to treat a variety of disorders in a multitargeting approach [43,44,45,46,47]. The most investigated compound, however, is SLC-0111 (U-104, Figure 1) [48,49,50,51,52,53] for the management of advanced, metastatic solid tumors; this compound is now in Phase Ib/II clinical trials [54].

For many years, especially CA IX and CA XII were in the focus of scientific interest to combat cancer. Recently, CA II in the endothelium of glial tumors became a potential target for therapy [55,56,57,58,59,60,61,62,63,64]. Furthermore, the CA II inhibitor acetazolamide was suggested as a chemosensitizer for treating temozolomide resistant gliomas [65,66,67,68,69]. In addition, CA II was found among up-regulated genes and as a candidate gene correlated with glioma malignancy and patient survival. [70]

The association of CA II with several pathological diseases prompted us to synthesize novel CA II inhibitors. Recently, it was revealed that methyl sulfamates, [71] derived from methyl triterpenoates such as oleanolic acid, ursolic acid, and glycyrrhetinic acid or betulinic acid, are effective and competitive inhibitors of CA II and several cycloartane derivatives [72] held significant activity for CA II, too. Euphol [73] was also shown to induce autophagy and to sensitize temozolomide cytotoxicity in glioblastoma cells. Several 1H-1,2,3-triazoles derived from pentacyclic triterpenoid 3-*O*-acetyl-(11-keto)-boswellic acids [74] were inhibitors CA II. Furthermore, hybrids of bile acids [75,76,77,78] have been shown to act as inhibitors of CAs.

**Figure 2:**
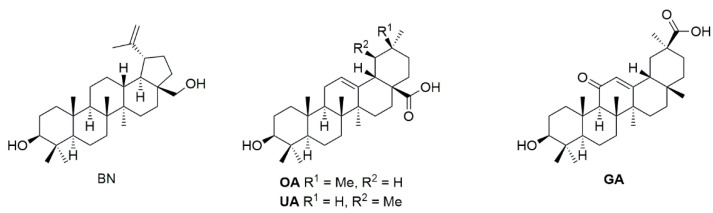
Structures of starting materials betulin (BN), oleanolic acid (OA), ursolic acid (UA), and glycyrrhetinic acid (GA).

Consequently, we became interested in the design, synthesis and CA II screening of conjugates derived from pentacyclic triterpenes such as betulin, oleanolic acid, ursolic acid, and glycyrrhetinic acid (Figure 2) with well-known inhibitor acetazolamide. 

## 2. Results

**Scheme 1:**
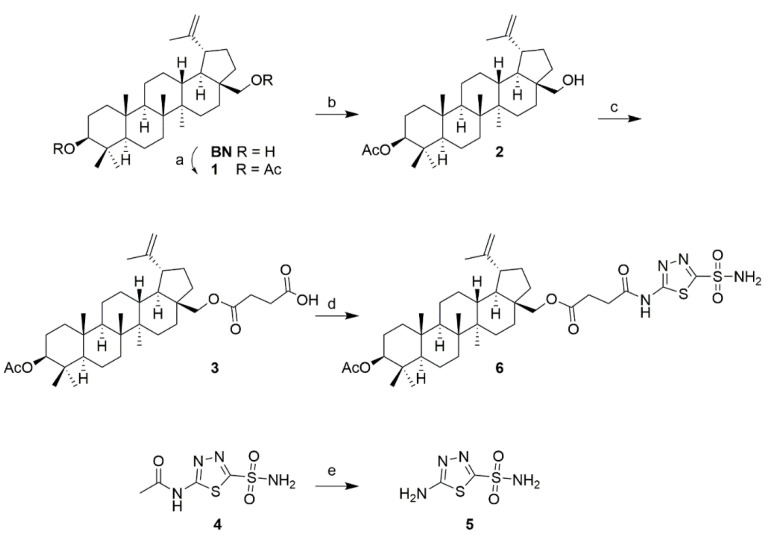
Reactions and conditions: (**a**) Ac_2_O, TEA, DMAP (cat.), DCM, 20 °C, 12 h, 90%; (**b**) CaH_2_, MeOH/THF, 20 °C, 12 h, 83%; (**c**) pyridine, DMAP, succinic anhydride, reflux, 15 h, 71%; (**d**) THF, 4-methyl-morpholine, ethyl chloroformate, 20 °C, 15 min, then **5**, reflux, 48 h, 88%; (**e**) conc. HCl, reflux, 3 h, 94%.

Based on our previous results [71], triterpenoids (Figure 2) betulin (**BN**), oleanolic acid (**OA**), ursolic acid (**UA**), and glycyrrhetinic acid (**GA**) appeared to us as suitable and representative starting materials. **BN** was converted (Scheme 1) to the diacetate **1** according to known procedures, the selective mono-deacylation of which with CaH_2_ in a methanol/water mixture gave the 3-*O*-acetate **2**. Reaction of **2** with succinic anhydride in pyridine in the presence of DMAP (cat.) furnished the succinyl derivative **3**.

Commercial acetazolamide (**4**) was deacetylated with conc. HCl under reflux and compound **5** was obtained. The reaction of **3** with **5** proved somewhat difficult in the beginning, as both an in situ generation of the corresponding carboxylic acid chloride and its coupling with **5** failed, as was the use of coupling reagents such as EDC, DCC, PyBOP, or T3P. However, a good yield of **6** was obtained by first reacting **3** with ethyl chloroformate in the presence of 4-methyl-morpholine in THF to yield a mixed anhydride in situ, whose reaction with **5** then gave 88% of **6**.

Compound **6** shows the basic carbon skeleton unchanged from the starting material **BN**, and it is further characterized by the presence of the acetyl group at position C-3 (^1^H NMR δ = 1.99 ppm, and in ^13^C NMR δ = 171.9 and 21.0 ppm). The succinyl spacer is characterized by the two CH_2_ groups (in ^13^C NMR at δ = 30.0 and 30.9 ppm). The heterocycle shows in its ^13^C NMR spectrum the characteristic signals at δ = 161.0 and 164.3 ppm; the sulfamate group held in the ^1^H NMR spectrum the signal for the NH_2_ group at δ = 8.30 ppm.

**Scheme 2:**
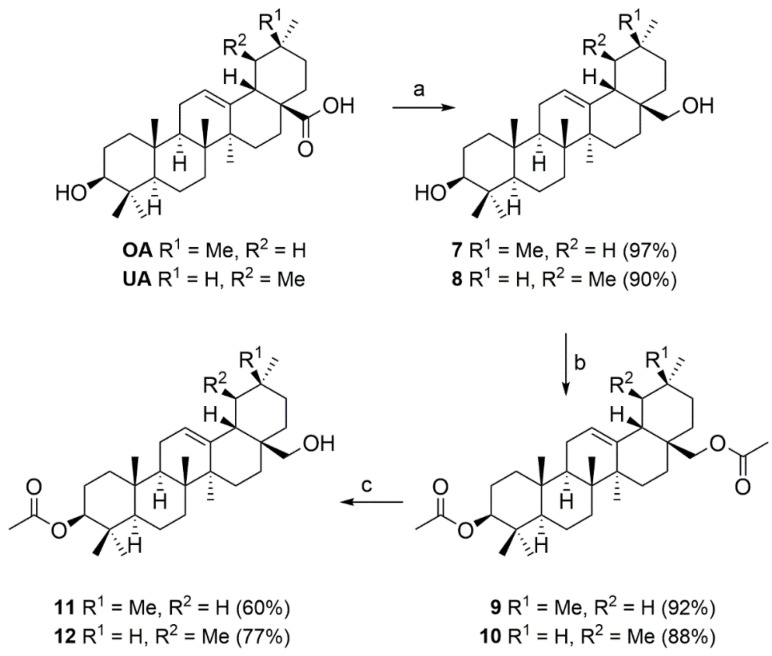
Reactions and conditions: (**a**) LiAlH_4_, THF, reflux, 2 h; (**b**) Ac_2_O, pyridine, 20 °C, 15 h; (**c**) Al(*i*PrO)_3_, *^i^*PrOH, reflux, 4 h.

For the preparation of the corresponding analogous compounds derived from **OA**, **UA**, or **GA**, the commercially relatively inexpensive triterpene carboxylic acids **OA**, **UA** first had to be reduced using LiAlH_4_ (Scheme 2). This allowed the corresponding diols **7** and **8** to be obtained in good yields. 

Compounds **7** and **8** were converted into the corresponding diacetates **9** and **10**, respectively, whose selective de-acetylation gave compounds **11** and **12**. Analogous conditions as described above could now be carried out for the subsequent reactions to yield the target compounds. 

**Scheme 3:**
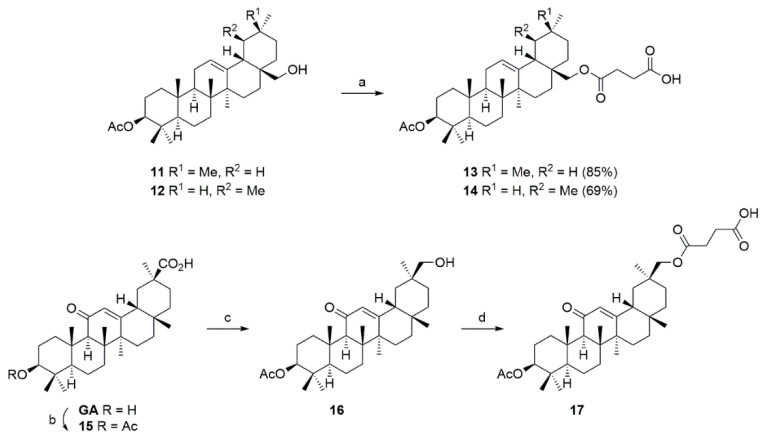
Reactions and conditions: (**a**) Pyridine, DMAP, succinic anhydride, reflux, 24 h; (**b**) Ac_2_O, pyridine, 20 °C, 15 h; (**c**) ethyl chloroformate, TEA, THF, −12 °C, 15 min, then sodium borohydride in water, 15 min; (**d**) pyridine, DMAP, succinic anhydride, reflux, 24 h.

Thus, the mono-acetates **11** and **12** were converted (Scheme 3) to the succinyl derivatives **13** and **14**.

Since the reduction of **GA** by LiAlH_4_ failed to give good yields, **GA** was first converted into acetate **15**, the reaction of which with ethyl chloroformate/TEA gave an un-isolated mixed anhydride, the reduction of which with NaBH_4_ at room temperature afforded compound **16** in good yields within a few minutes. Its reaction with succinyl anhydride yielded **17**.

**Scheme 4:**
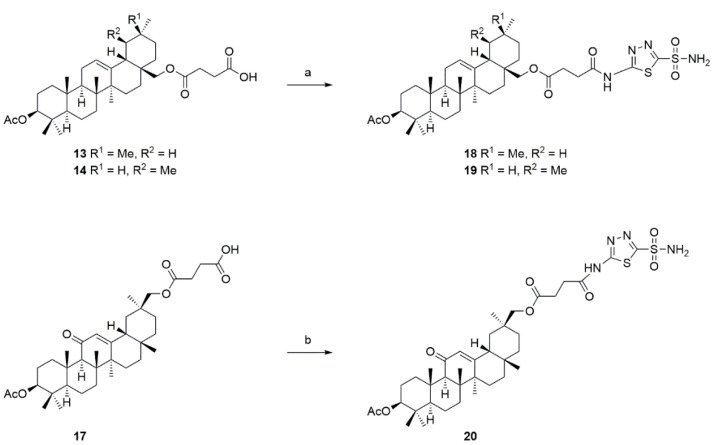
Reactions and conditions: (**a**,**b**) THF, 4-methyl-morpholine, ethyl chloroformate, 20 °C, 15 min, then **5**, reflux, 48 h.

The coupling of **13**, **14,** and **17** with **5** (Scheme 4) gave the products **18**–**20**, respectively.

Screening of compounds **6**, **18**–**20** for their activity was performed with CA II as previously described; the results from the assays are compiled in Table 1. Acetazolamide (**4**) was used as a positive control.

These assays showed glycyrrhetinic acid-derived conjugate **20** as the best inhibitor for this enzyme followed by betulin-derived **6**. These compounds were even better inhibitors than gold standard acetazolamide (**4**). Oleanolic and ursolic-derived conjugates showed a diminished ability to inhibit CA II. Parent compounds, i.e., betulin, betulinic acid, ursolic acid, oleanolic acid, and glycyrrhetinic acid did not inhibit the enzyme under the conditions of the assay at all. Compounds **2**, **3**, **7**–**17** showed inhibition rates less than 10%.

**Figure 3:**
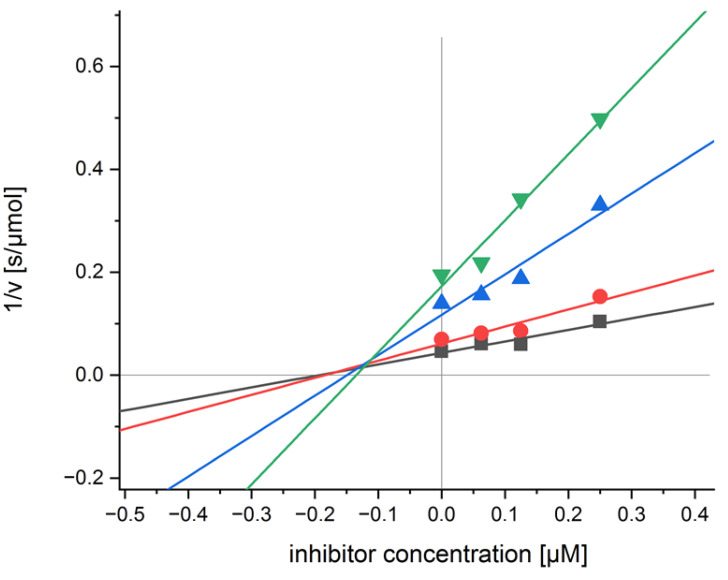
Dixon plot for compound **6** and CA II.

For compounds with the highest inhibition percentage, i.e., **6** and **19** and **20**, some extra measurements were performed to determine their respective inhibition constants K_i_ values. The results from these experiments are summarized in Table 2; Figure 3 shows the Dixon plot for compound **6**; this compound acts as a competitive inhibitor for the enzyme and holds a rather low K_i_ = 0.129 μM.

Initial molecular modelling calculations were performed to get some insights in the mode of action of the conjugates. These calculations, however, did not provide any reasonable explanation for the different ability of the conjugates to inhibit the enzyme. While it seems plausible that the acetazolamide moiety interacts with the active site of the enzyme in a manner like parent acetazolamide, it cannot be excluded; however, that the conjugates also act as non-zinc binding inhibitors, thus paralleling previous findings for structurally similar pentacyclic triterpenoid arjunolic acid [79]. 

Previously especially CA IX was extensively studied in the process of tumorigenesis, [15,80] and several derivatives of pentacyclic triterpenoids have been revealed as inhibitors of this isoform, too [81]. The selectivity of the triterpenoid investigated so far toward individual isoforms of CA, however, was not particularly pronounced.

Compounds **6** and **18**–**20** were screened for their cytotoxic activity in sulforhodamine B assays (SRB), employing several human tumor cell lines. The results from these assays are summarized in Table 3. Expression of CA II and its involvement cancer has previously been established for A375 [82], HT29 [83] as well as for MCF-7 cells [84]. Cell line A2780 and non-malignant fibroblasts (NIH 3T3) were employed for comparison.

As a result, the highest cytotoxicity was established for botulin-derived **6** followed by glycyrrhetinic acid-derived **20**. This parallels the finding for the inhibition rates for CA II established for these compounds. A significantly lower cytotoxicity was determined for oleanolic or ursolic acid-derived compounds **18** and **19**, respectively. The malignant/non-malignant cell selectivity, however, was low for all compounds. No cytotoxicity (EC_50_ > 30 μM; cut-off of the assay) was found for parent triterpenoic acids.

## 3. Conclusions

Pentacyclic triterpenoids betulin, oleanolic acid, ursolic acid, and glycyrrhetinic acid were acetylated at position C-3 and converted into their succinyl-spacered acetazolamide conjugates. Their screening for their inhibitory activity onto carbonic anhydrase II and screening for their cytotoxicity in SRB assays employing several human tumor cell lines and non-malignant fibroblasts showed the conjugates derived from betulin and glycyrrhetinic acid to be the best inhibitors while those derived from ursolic or oleanolic acid were significantly weaker inhibitors but also of diminished cytotoxicity. A botulin-derived conjugate held a K_i_ = 0.129 μM and an EC_50_ = 8.5 μM for human A375 melanoma cells.

## 4. Experimental

NMR spectra were recorded using the Varian spectrometers (Darmstadt, Germany) DD2 and VNMRS (400 and 500 MHz, respectively). MS spectra were taken on a Advion expressionL CMS mass spectrometer (Ithaca, USA; positive ion polarity mode, solvent: methanol, solvent flow: 0.2 mL/min, spray voltage: 5.17 kV, source voltage: 77 V, APCI corona discharge: 4.2 μA, capillary temperature: 250 °C, capillary voltage: 180 V, sheath gas: N2). Thin-layer chromatography was performed on pre-coated silica gel plates supplied by Macherey-Nagel (Düren, Germany). IR spectra were recorded on a Spectrum 1000 FT-IR-spectrometer from Perkin Elmer (Rodgau, Germany). The UV/Vis-spectra were recorded on a Lambda 14 spectrometer from Perkin Elmer (Rodgau, Germany); optical rotations were measured using a JASCO-P2000 instrument (JASCO Germany GmbH, Pfungstadt, Germany) The melting points were determined using the Leica hot stage microscope Galen III (Leica Biosystems, Nussloch, Germany) and are uncorrected. The solvents were dried according to usual procedures. Microanalyses were performed with an Elementar Vario EL (CHNS) instrument (Elementar Analysensysteme GmbH, Elementar-Straße 1, D-63505 Langenselbold, Germany). All dry solvents were distilled over respective drying agents except for DMF which was distilled and stored under argon and molecular sieve. Reactions using air- or moisture-sensitive reagents were carried out under argon atmosphere in dried glassware. Triethylamine was stored over potassium hydroxide. Biological assays were performed as previously reported employing cell lines obtained from the Department of Oncology [Martin-Luther-University Halle Wittenberg; they were bought from ATCC: malignant: A 375, HT29, MCF7, and A2780; non-malignant: NIH 3T3]. Oleanolic and ursolic acid were obtained from Betulinines (Strbrna Skalice, Czech Republic) and used as received. Glycyrrhetinic acid was bought from Orgentis Chemicals GmbH (Gatersleben).

For the SRB assay: cells were seeded into 96-well plates on day zero at appropriate cell densities to prevent confluence of the cells during the period of the experiment. After 24 h, the cells were treated with different concentrations (1, 3, 7, 12, 20, and 30 μM), but the final concentration of DMSO/DMF never exceeded 0.5%, which was non-toxic to the cells. After 72 h of treatment, the supernatant media from the 96-well plates were discarded, then the cells were fixed with 10% trichloroacetic acid and allowed to rest at 4 °C. After 24 h of fixation, the cells were washed in a strip washer and then dyed with SRB solution (200 μL, 10 mM) for 20 min. Then the plates were washed four times with 1% acetic acid to remove the excess of the dye and allowed to air-dry overnight. Tris base solution (200 μL, 10 mM) was added to each well. The absorbance was measured with a 96-well plate reader from Tecan Spectra. 

For the CA II assay: Carbonic anhydrase II (bCA II, ≥3000 W-A units/mg from bovine erythrocytes) as well as 4-nitrophenyl acetate (4-NA) were purchased from Sigma.

A 96-well microplate spectrometer BMG Labtech Spectrostar Omega working in the slow kinetics mode and measuring the absorbance at a distinct wavelength of λ = 415 nm with center scanning was used for the enzymatic studies. In short: A mixture of 4-NA solution (125 µL, 6 mM in 50 mM Tris-HCl buffer, pH 8), enzyme solution (25 µL, 0.3 mg/mL), and compounds solutions (25 µL, 3 different concentrations and water as a blank) was incubated at 37 °C for 20 min. The substrate (25 µL, [4-NA] = 0.75 mM, 0.50 mM, 0.25 mM, 0.15 mM) was added to start the enzymatic reaction. The absorbance data was recorded under a controlled temperature of 37 °C for 30 min at 1 min intervals at λ = 415 nm. The relative inhibition was determined as the quotient of the slopes (compound divided by blank) of the linear ranges.

### 4.1. Di-O-Acetyl-betulin

Compound 1 was prepared from BN (15.0 g, 34 mmol) by acetylation with acetic anhydride as previously described; re-crystallization from ethanol gave 1 (15.8 g, 90%) as a white solid; R*_F_* = 0.73 (silica gel, hexanes/ethyl acetate, 8:2); m.p.: 221 °C (lit.: [85] 216–218 °C); [α]_D_ = +16.6° (*c* = 0.061, MeOH), [lit.: [85] [α]_D_ = +19.7° (CHCl_3_)]; MS (ESI, MeOH): *m*/*z* = 467.5 (100%, [M + H-HOAc]^+^).

### 4.2. 3-O-Acetyl-betulin

Selective deacetylation of 1 (8.0 g, 15.2 mmol) with cat. amounts of CaH_2_ in MeOH/THF (100 mL, 1:1 *v:v*) for 12 h at 20 °C followed by usual aqueous workup and chromatography (silica gel, hexanes/ethyl acetate, 8:2) gave 2 (6.1 g, 83%) as a colorless solid; R*_F_* = 0.40 (silica gel, hexanes/ethyl acetate, 8:2); m.p.: 256–259 °C (lit.: [85] 258–260 °C); [α]_D_ = +28.8° (*c* = 0.039, CHCl_3_), [lit.: [85] [α]_D_ = +25.7° (CHCl_3_)]; MS (ESI, MeOH): *m*/*z* = 992,0 (100%, [2M+Na]^+^).

### 4.3. 4-{[(3β)-3-(Acetyloxy)lup-20(29)-en-28-yl]oxy}-4-oxobutanoic Acid

To a solution of 2 (4.0 g, 8.2 mmol) in dry pyridine (50 mL), DMAP (cat.) and succinic anhydride (1.64 g, 16.4 mmol) were added. The reaction mixture was stirred under reflux for 15 h. Usual aqueous work up and chromatography (silica gel, hexanes/ethyl acetate, 7:3) gave 3 [86,87,88,89,90,91] (3.4 g, 71%) as a white solid; m.p. 189–191 °C (lit.: [86,87,88] 190–191 °C); [α]_D_ = +12.1° (*c* = 0.198, MeOH); R_F_ = 0.15 (silica gel, hexanes/ethyl acetate, 8:2); IR (ATR): ν = 2943*m*, 2871*w*, 1732*s*, 1713*s*, 1455*w*, 1361*w*, 1366*m*, 1244*s*, 1159*m*, 1027*w*, 979*m*, 883*w*, 754*m* cm^−1^; ^1^H NMR (500 MHz, CDCl_3_): δ = 4.68 (s, 1H, 29-H_a_), 4.58 (s, 1H, 29-H_b_), 4.46 (dd, J = 10.6, 5.6 Hz, 1H, 3-H_a_), 4.30 (d, J = 11.0 Hz, 1H, 28-H_a_), 3.88 (d, J = 11.1 Hz, 1H, 28-H_b_), 2.75–2.54 (m, 4H, 34-H, 35-H), 2.42 (td, J = 11.0, 5.8 Hz, 1H, 19-H), 2.03 (s, 3H, 32-H), 2.01–1.88 (m, 1H, 21-H_a_), 1.85–1.79 (m, 1H, 16-H_a_), 1.75 (dd, J = 12.5, 7.9 Hz, 1H, 22-H_a_), 1.68 (s, 3H, 30-H), 1.72–1.54 (m, 7H, 1-H_a_, 13-H, 15-H_a_, 12-H_a_, 2-H, 9-H), 1.50 (s, 1H, 6-H_a_), 1.44–1.35 (m, 5H, 6-H_b_, 11-H_a_, 21-H_b_, 7-H), 1.34–1.14 (m, 3H, 16-H_b_, 18-H, 11-H_b_), 1.02 (s, 3H, 23-H), 1.12–0.90 (m, 4H, 22-H_b_, 12-H_b_, 15-H_b_, 1-H_b_), 0.96 (s, 3H, 27-H), 0.84 (s, 3H, 24-H), 0.84 (s, 3H, 26-H), 0.83 (s, 3H, 25-H), 0.78 (m, 1H, 5-H). ppm; ^13^C NMR (126 MHz, CDCl_3_): δ = 177.8 (C-36), 172.6 (C-33), 171.3 (C-31), 150.2 (C-20), 110.0 (C-29), 81.1 (C-3), 63.3 (C-28), 55.5 (C-5), 50.4 (C-18), 48.9 (C-9), 47.9 (C-19), 46.6 (C-17), 42.8 (C-14), 41.0 (C-8), 38.5 (C-1), 37.9 (C-4), 37.7 (C-13), 37.2 (C-10), 34.2 (C-22), 29.9 (C-16), 29.1 (C-35), 28.1 (C-24), 27.2 (C-15), 25.3 (C-12), 23.8 (C-2), 21.4 (C-32), 20.9 (C-11), 19.3 (C-30), 18.3 (C-6), 16.6 (C-25), 16.2 (C-23), 14.9 (C-27) ppm; MS (ESI, MeOH): *m/z* = 583.6 (100%, [M-H]^-^); analysis calcd for C_36_H_56_O_6_ (584.83): C 73.93, H 9.65; found: C 73.67, H 9.88.

### 4.4. 5-Amino-1,3,4-thiadiazole-2-sulfonamide

A solution of acetazolamide (4, 9.0 g, 40.7 mmol) in conc. HCl (60 mL) was heated under reflux for 3 h. After neutralization with NaOH, saturation with NaCl and extraction with THF (4 × 100 mL) followed by removal of the organic solvent, 5 (6.9 g, 94 %) was obtained as a white solid; m.p. 195 °C decomp. (lit.: [92] 215.5–216); R_F_ = 0.3 (silica gel, CHCl_3_/MeOH, 9:1); UV-Vis (MeOH): λ_max_ (log ε) = 278 nm (3.80) IR (ATR): ν = 3427*w*, 3321*m*, 3173*w*, 2870*w*, 2636*w*, 1601*s*, 1496*s*, 1448*m*, 1338*s*, 1172*m*, 1139*m*, 1098*w*, 1058*w*, 941*m*, 647*s*, 581*s*, 484*w* cm^−1^; ^1^H NMR (500 MHz, DMSO-*d*_6_): δ = 8.04 (s, 2H, NH_2_), 7.84 (s, 2H, NH_2_) ppm; ^13^C NMR (126 MHz, DMSO-*d*_6_): δ = 171.7, 157.9. ppm; MS (ESI, MeOH): *m/z* = 179.0 (100%, [M-H]^-^).

### 4.5. (3β)-3-(Acetyloxy)lup-20(29)-en-28-yl 4-{[5-(aminosulfonyl)-1,3,4-thiadiazol-2-yl]amino}-4-oxobutanoate

Compound 3 (500 mg, 0.85 mmol) was dissolved in dry THF (50 mL), 4-methylmorpholine (172 mg, 1.7 mmol) and ethyl chloroformate (185 mg, 1.7 mmol.) were added. The reaction mixture was stirred at 20 °C for 15 min. Compound 5 (184 mg, 1.02 mmol) was added, and the mixture was heated under reflux for another 48 h. The solvent was removed, the residue dissolved in CHCl_3_, washed with 2 M NaOH, water and brine and dried (MgSO_4_). Chromatography (silica gel, CHCl_3_/MeOH, 9:1) gave 4 (560 mg, 88%) as a white solid; m.p. 161–164°C; R_F_ = 0.55 (silica gel, hexanes/ethyl acetate, 7:3); UV-Vis (CHCl_3_): λ_max_ (log ε) = 264 nm (3.92) IR (ATR): ν = 2944*m*, 1733*m*, 1701*m*, 1531*w*, 1371*m*, 1245*s*, 1173*s*, 1018*w,* 979*m*, 882*w*, 609*m*, 504*w* cm^−1^; ^1^H NMR (500 MHz, DMSO-*d*_6_): δ = 8.30 (s, 2H, NH_2_), 4.69 (s, 1H, 29-H_a_), 4.55 (s, 1H, 29-H_b_), 4.36 (dd, *J* = 11.4, 4.7 Hz, 1H, 3-H), 4.23 (d, *J* = 10.9 Hz, 1H, 28-H_a_), 3.78 (d, *J* = 11.1 Hz, 1H, 28-H_b_), 2.87–2.78 (m, 2H, 35-H), 2.76–2.65 (m, 2H, 34-H), 2.43 (s, 1H, 19-H), 1.99 (s, 3H, 32-H), 1.85 (m, 1H, 21-H_a_), 1.63 (s, 3H, 30-H), 1.76–1.43 (m, 9H, 16-H_a_, 22-H_a_, 12-H_a_, 13-H, 1-H_a_, 9-H, 15-H_a_, 2-H), 1.42–1.12 (m, 9H, 6-H, 11-H_a_, 21-H_b_, 7-H, 18-H, 16-H_b_, 11-H_b_), 0.95 (s, 3H, 23-H), 0.93 (s, 3H, 27-H), 1.09–0.73 (m, 5H, 22-H_b_, 12-H_b_, 1H_b_, 15-H_b_, 5-H), 0.79 (s, 9H, 24-H, 25-H, 26-H) ppm; ^13^C NMR (126 MHz, DMSO-*d*_6_): δ = 171.9 (C-31), 171.3 (C-33), 170.1 (C-36), 164.3 (C-38), 161.0 (C-37), 149.8 (C-20), 110.0 (C-29), 79.9 (C-3), 61.9 (C-28), 54.6 (C-5), 49.5 (C-18), 48.1 (C-9), 47.0 (C-19), 46.0 (C-17), 42.2 (C-14), 40.4 (C-8), 37.4 (C-4), 37.0 (C-13), 36.6 (10), 34.2, 34.0 (22), 33.5 (7), 30.9 (34), 30.0 (35), 29.1 (16), 28.9, 28.5 (21), 27.6 (24), 26.6 (C-15), (C-12), 23.4 (C-2), 21.0 (C-32), 20.3 (C-11), 18.7 (C-30), 17.7 (C-6), 16.4 (C-25), 15.8 (C-26), 15.5 (C-23), 14.5 (C-27) ppm; MS (ESI, MeOH): *m/z* = 745.7 (100%, [M-H]^-^); analysis calcd for C_38_H_58_N_4_O_7_S_2_ (747.03): C 61.10, H 7.83, N 7.50; found: C 60.85, H 8.03, N 7.33.

### 4.6. (3β) Olean-12-en3-3,28-diol (Erythrodiol)

To a solution of OA (5.0 g, 10.7 mmol, 1.00 eq.) in dry THF (150 mL), LiAlH_4_ (2.0 g, 53.6 mmol, 5.00 eq.,) was slowly added. Stirring under reflux was continued for another 2 h. After cooling to 20 °C, the reaction was quenched (slow addition of 20 mL MeOH), and aq. HCl (6 M, 50 mL) was added. The reaction mixture was extracted with ethyl acetate (3 × 75 mL); the combined organic phases were washed with aq. NaOH (1 M, 2 × 50 mL), brine (50 mL) and dried (MgSO_4_). The solvent was removed under reduced pressure, and the residue was subjected to chromatography (silica gel, chloroform/hexanes/ethyl acetate, 10:8:2) to yield 7 (4.61 g, 97%) as a colorless solid; m.p. 218–219 °C (lit.: [93] 217–219 °C); R_F_ = 0.17 (silica gel, chloroform/hexanes/ethyl acetate, 10:8:2); [α]_D_ = +72.1° (*c* = 0.113, MeOH) (lit.: [94] [α]_D_ = +75.0° (*c* = 0.325, CHCl_3_)).

### 4.7. (3β) Urs-12-ene-3,28-diol (Uvaol, 8)

Following the procedure given for the synthesis of 7, from UA (5.00 g, 10.7 mmol), dry THF (150 mL) and LiAlH_4_ (2.0 g, 53.6 mmol) followed by chromatography (silica gel, (chloroform/hexanes/ethyl acetate, 10:8:2) 8 (4.36 g, 90%) was obtained as a colorless solid; m.p. 227–229 °C (lit.: [93] 225–227 °C); R_F_ = 0.18 (silica gel, chloroform/hexanes/ethyl acetate, 10:8:2); [α]_D_ = +60.5° (*c* = 0.109, MeOH); (lit.: [95] [α]_D_ = +62.6° (*c* = 0.62, CHCl_3_)).

### 4.8. (3β)-Olean-12-ene-3,28-diyl Diacetate

Acetylation of 7 (4.00 g, 9.04 mmol) in dry pyridine (16 mL) with acetic anhydride (2.6 mL, 27.1 mmol) for 15 h at 20 °C followed by usual aqueous work up and chromatography (silica gel, (hexanes/ethyl acetate, 9:1) gave 9 (4.37 g, 92%) as a colorless solid; m.p. 184-186 °C (lit.: [96] 184-186 °C); R_F_ = 0.43 (silica gel, hexanes/ethyl acetate, 9:1); [α]_D_ = +62.2° (*c* = 0.124, CHCl_3_); (lit.: [97] [α]_D_ = +56.0° (*c* = 1.0CHCl_3_)).

### 4.9. (3β)-Urs-12-ene-3,28-diyl Diacetate

Acetylation of 8 (3.48 g, 7.86 mmol) as described above for the synthesis of 9 gave 10 (3.66 g, 88%) as a colorless solid; m.p. 151–153 °C (lit.: [98] 150-151 °C); R_F_ = 0.41 (silica gel, hexanes/ethyl acetate, 9:1); [α]_D_ = +51.4° (*c* = 0.115, CHCl_3_). 

### 4.10. (3β)-28-Hydroxyolean-12-en-3-yl Acetate

A solution of 9 (3.1 g, 5.89 mmol) and aluminum isopropoxide (12.3 g, 58.8 mmol) in isopropanol (150 mL) was heated under reflux for 4 h. Usual aqu. work-up followed by chromatography (silica gel, hexanes/ethyl acetate, 8:2) gave 11 (1.71 g, 60%) as a colorless solid; m.p. 234–236 °C (lit.: [99] 233–234 °C); R_F_ = 0.55 (silica gel, hexanes/ethyl acetate, 8:2); [α]_D_ = +67.4° (*c* = 0.122, CHCl_3_) (lit.: [100] [α]_D_ = +71° (*c* = 0.70, CHCl_3_)).

### 4.11. (3β)-28-Hydroxyolean-12-en-3-yl Acetate

As described above for the synthesis of 11, from 10 (2.7 g, 5.13 mmol) and aluminum propoxide (10.7 g, 51.3 mmol) in isopropanol (150 mL) followed by chromatography (silica gel, hexanes/ethyl acetate, 8:2) 12 (1.92 g, 77%) was obtained as a colorless solid; m.p. 265–267 °C (lit.: [101] 258–261 °C); R_F_ = 0.51 (silica gel, hexanes/ethyl acetate, 8:2); [α]_D_ = +63.1° (*c* = 0.139, CHCl_3_) (lit.: [102] [α]_D_ = +70.5° (*c* = 0.145, CHCl_3_)).

### 4.12. 4-{[(3β)-3-(Acetyloxy)-olean-12-en-28-yl]oxy}-4-oxobutanoic Acid

To a solution of 11 (0.45 g, 0.928 mmol) in dry pyridine (15 mL) succinic anhydride (0.188 g, 1.86 mmol) and cat. DMAP were added, and the mixture was stirred for 1 day under reflux. Usual aq. work-up followed by chromatography (silica gel, hexanes/ethyl acetate (1% HCOOH), 8:2 → 7:3) gave 13 (0.460 g, 85%) as a colorless solid; m.p. 124–126 °C; R_F_ = 0.47 (silica gel, hexanes/ethyl acetate, 7:3); [α]_D_ = +49.6° (*c* = 0.126, CHCl_3_); IR (ATR): ν = 2946*m*, 2864 *w*, 1734*s*, 1712*s*, 1463*w*, 1432*w*, 1387*m*, 1364*m*, 1244*s*, 1160*s*, 1095*w*, 1027*m*, 1004*m*, 986*m*, 967*m* cm^−1^; ^1^H NMR (500 MHz, CDCl_3_): δ = 8.01 (brs, 1H, COO-H), 5.19 (t, *J* = 3.6 Hz, 1H, 12-H), 4.55–4.44 (m, 1H, 3-H), 4.07 (d, *J* = 11.0 Hz, 1H, 28-H_a_), 3.72 (d, *J* = 11.0 Hz, 1H, 28-H_b_), 2.71–2.59 (m, 4H, 34-H + 35-H), 2.04 (s, 3H, 32-H), 2.01–1.99 (m, 1H, 18-H), 1.97–1.78 (m, 3H, 16-H_a_ + 11-H), 1.77–1.46 (m, 9H, 19-H_a_ + 15-H_a_ + 2-H + 1-H_a_ + 9-H + 6-H_a_ + 7-H_a_ + 22-H_a_), 1.45–1.21 (m, 4H, 6-H_b_ + 22-H_b_ + 7-H_b_ + 21-H_a_), 1.19–1.10 (m, 5H, 16-H_b_ + 21-H_b_ + 27-H), 1.12–0.95 (m, 3H, 19-H_b_ + 1-H_b_ + 15-H_b_), 0.94 (s, 3H, 25-H), 0.93 (s, 3H, 26-H), 0.88 (s, 3H, 30-H), 0.86 (s, 6H, 29-H + 24-H), 0.85 (s, 3H, 23-H), 0.84–0.80 (m, 1H, 5-H) ppm; ^13^C NMR (126 MHz, CDCl_3_): δ = 177.9 (C-36), 172.2 (C-33), 171.3 (C-31), 143.7 (C-13), 123.0 (C-12), 81.1 (C-3), 71.3 (C-28), 55.4 (C-5), 47.6 (C-9), 46.3 (C-19), 42.7 (C-18), 41.8 (C-14), 39.9 (C-8), 38.4 (C-1), 37.8 (C-4), 36.9 (C-10), 36.0 (C-17), 34.1 (C-21), 33.3 (C-30), 32.6 (C-7), 31.6 (C-22), 31.0 (C-20), 29.1 (C-34), 29.1 (C-35), 28.2 (C-24), 26.1 (C-27), 25.7 (C-15), 23.7 (C-29), 23.7 (C-11), 23.7 (C-2), 22.3 (C-16), 21.4 (C-32), 18.4 (C-6), 16.8 (C-26), 16.8 (C-23), 15.7 (C-25) ppm; MS (ESI, MeOH/CHCl_3_, 4:1): *m/z* = 583.9 (100%, [M-H]^-^); analysis calcd for C_36_H_56_O_6_ (584.84): C 73.93, H 9.65; found: C 73.71, H 9.86.

### 4.13. 4-{[(3β)-3-(Acetyloxy)urs-12-en-28-yl]oxy}-4-oxobutanoic Acid

Following the procedure given for 11, from 12 (1.35 g, 2.79 mmol), 14 (1.12 g, 69%) was obtained as a colorless solid; m.p. 112–114 °C; R_F_ = 0.50 (silica gel, hexanes/ethyl acetate, 7:3); [α]_D_ = +42.7° (*c* = 0.131, CHCl_3_); IR (ATR): ν = 2948*m*, 2925*m*, 1734*s*, 1712*s*, 1456*m*, 1432*w*, 1388*m*, 1370*m*, 1269*m*, 1244*s*, 1158*s*, 1095*w*, 1025*m*, 1006*m*, 985*m*, 967*m* cm^−1^; ^1^H NMR (500 MHz, CDCl_3_): δ = 8.01 (*brs*, 1H, COO-H), 5.13 (dd, *J* = 3.7 Hz, 1H, 12-H), 4.53–4.44 (m, 1H, 3-H), 4.10 (d, *J* = 11.0 Hz, 1H_,_ 28-H_a_), 3.64 (d, *J* = 11.0 Hz, 1H, 28-H_b_), 2.70–2.60 (m, 4H, 34-H + 35-H), 2.04 (s, 3H, 32-H), 1.98–1.88 (m, 2H, 16-H_a_ + 11-H), 1.76 – 1.68 (m, 1H, 15-H_a_), 1.67–1.59 (m, 2H, 1-H_a_ + 2-H), 1.59–1.48 (m, 4H, 22-H_a_ + 7-H_a_ + 9-H + 6-H_a_), 1.47–1.28 (m, 6H, 21-H_a_ + 18-H + 6-H_b_ + 19-H + 7-H_b_ + 22-H_b_), 1.27–1.13 (m, 2H, 21-H_b_ + 16-H_b_), 1.08 (s, 3H, 27-H), 1.08–1.06 (m, 1H, 1-H_b_), 0.99–0.96 (m, 1H, 15-H_b_), 0.97 (s, 3H, 26-H), 0.96 (s, 3H, 25-H), 0.93 (d, *J* = 5.8 Hz, 3H, 29-H), 0.91–0.88 (m, 1H, 20-H), 0.86 (s, 3H, 24-H), 0.86 (s, 3H, 23-H), 0.85–0.83 (m, 1H, 5-H), 0.80 (d, *J* = 5.3 Hz, 3H, 30-H) ppm; ^13^C NMR (126 MHz, CDCl_3_): δ = 178.0 (C-36), 172.2 (C-33), 171.3 (C-31), 138.3 (C-13), 125.7 (C-12), 81.1 (C-3), 71.8 (C-28), 55.4 (C-5), 54.4 (C-18), 47.7 (C-9), 42.1 (C-14), 40.1 (C-8), 39.5 (C-20), 39.3 (C-19), 38.6 (C-1), 37.8 (C-17), 37.1 (C-4), 36.9 (C-10), 35.8 (C-22), 32.8 (C-7), 30.6 (C-21), 29.2 (C-34), 29.2 (C-35), 28.2 (C-24), 26.1 (C-15), 23.7 (C-2), 23.5 (C-11), 23.5 (C-16), 23.5 (C-27), 21.4 (C-32), 21.4 (C-29), 18.3 (C-6), 17.4 (C-30), 16.9 (C-26), 16.8 (C-23), 15.9 (C-25) ppm; MS (ESI, MeOH/CHCl_3_, 4:1): *m/z* = 607.9 (100%, [M + Na]^+^); analysis calcd for C_36_H_56_O_6_ (584.84): C 73.93, H 9.65; found: C 73.68, H 9.91.

### 4.14. (3β, 20β)-3-Acetyloxy-11-oxoolean-12-en-29-oic Acid

Acetylation of GA (2.50 g, 5.31 mmol) as described above followed by chromatography (silica gel, hexanes/ethyl acetate, 8:2) gave 15 (2.15 g, 79%) as a colorless solid; m.p. 305–307 °C (lit.: [96] 316–318 °C); R_F_ = 0.41 (silica gel, hexanes/ethyl acetate, 9:1); [α]_D_ = +163.3° (*c* = 0.142, CHCl_3_); (lit.: [103] [α]_D_ = +165.1° (*c* = 0.7, CHCl_3_)). 

### 4.15. (3β, 20β) 3-Acetyloxy-29-hydroxyolean-12-en-11-one

To a solution of 15 (1.3 g, 2.76 mmol) and triethylamine (1.1 mL, 7.60 mmol) in dry THF (15 mL), at −12 °C, ethyl chloroformate (1.1 mL, 11.1 mmol) was added, and the mixture was stirred for 15 min. The precipitate was filtered off, and the filtrate was slowly added to a freshly prepared solution of sodium borohydride (0.522 g, 13.8 mol) in water (2.5 mL). Stirring at room temperature was continued for another 15 min followed by usual aq. work-up and chromatography (silica gel, CHCl_3_/Et_2_O/hexanes/HCOOH, 25:25:43:7) to yield 16 (1.09 g, 82%) as a colorless solid; m.p. 264–266 °C; R_F_ = 0.46 (silica gel, CHCl_3_/Et_2_O/hexanes/HCOOH, 25:25:43:7); [α]_D_ = +91.6° (*c* = 0.129, CHCl_3_); UV-Vis (CHCl_3_): λ_max_ (log ε) = 249 nm (4.07); IR (ATR): ν = 3569*w*, 3550 *w*, 2925*m*, 2862*w*, 1725*s*, 1695*m*, 1651*s*, 1626*m*, 1465*w*, 1455*m*,1386*m*, 1366*m*, 1325*w*, 1279*w*, 1246*s*, 1209*m*, 1173*s*, 1143*m*, 1095*w*, 1048*m*, 1025*s*, 1001*m*, 985*m* cm^−1^; ^1^H NMR (500 MHz, CDCl_3_): δ = 5.58 (s, 1H, 12-H), 4.50 (dd, *J* = 11.8, 4.7 Hz, 1H, 3-H), 4.13 (d, *J* = 11.0 Hz, 1H_,_ 30-H_a_), 4.03 (d, *J* = 11.0 Hz, 1H, 30-H_b_ (30)), 2.78 (dt, *J* = 13.6, 3.6 Hz, 1H, 1-H_a_), 2.35 (s, 1H, 9-H), 2.13–2.06 (m, 2H, 18-H + 16-H_a_), 2.04 (s, 3H, 32-H), 1.86–1.76 (m, 1H, 15-H_a_), 1.74–1.53 (m, 7H, 2-H_a_ + 22-H_a_ + 7-H_a_ + 19-H_a_ + 2-H_b_ + 21-H_a_ + 6-H_a_) 1.50–1.36 (m, 3H, 6-H_b_ + 7-H_b_ + 22-H_b_ + 19-H_b_), 1.36 (s, 3H, 27-H), 1.21– 1.16 (m, 1H, 15-H_b_), 1.15 (s, 3H, 25-H), 1.12 (s, 3H, 26-H), 1.07–0.98 (m, 2H, 1-H_b_ + 16-H_b_), 0.95 (s, 3H, 29-H), 0.87 (s, 6H, 28-H + 23-H), 0.86 (s, 3H, 24-H), 0.81–0.77 (m, 1H, 5-H), 0.80 (d, *J* = 5.7 Hz, 3H, 30-H) ppm; ^13^C NMR (126 MHz, CDCl_3_): δ = 200.4 (C-11), 171.4 (C-31), 169.8 (C-13), 128.5 (C-12), 80.9 (C-3), 67.0 (C-30), 61.8 (C-9), 55.2 (C-5), 47.0 (C-18), 45.6 (C-8), 43.5 (C-14), 40.2 (C-19), 38.9 (C-1), 38.2 (C-4), 37.1 (C-10), 36.0 (C-22), 34.3 (C-20), 32.8 (C-7), 32.4 (C-17), 30.2 (C-21), 28.6 (C-28), 28.2 (C-24), 27.9 (C-29), 26.7 (C-16), 26.5 (C-15), 23.7 (C-2), 23.5 (C-27), 21.4 (C-32), 18.8 (C-26), 17.5 (C-6), 16.8 (C-23), 16.5 (C-25) ppm; MS (ESI, MeOH/CHCl_3_, 4:1): *m/z* = 497.9 (100%, [M-H]^-^); analysis calcd for C_32_H_50_O_4_ (498.74): C 77.06, H 10.10; found: C 76.81, H 10.35.

### 4.16. 4-{[(3β,20β)3-(Acetyloxy)-11-oxoolean-12-en-30-yl]oxy}-4-oxobutanoic Acid

Following the procedure described above, from 16 (0.270 g, 0.541 mmol) 17 (0.315 g, 97%) was obtained as a colorless solid; m.p. 109–111 °C; R_F_ = 0.44 (silica gel, hexanes/ethyl acetate, 1:1); [α]_D_ = +110.5° (*c* = 0.118, CHCl_3_); UV-Vis (CHCl_3_): λ_max_ (log ε) = 254 nm (4.05); IR (ATR): ν = 2949*m*, 2928*m*, 2871*w*, 1730*s*, 1657*m*, 1465*w*, 1456*w*, 1388*m*, 1365*m*, 1322*w*, 1244*s*, 1209*m*, 1158*m*, 1091*w*, 1049*w*, 1028*m*, 1001*m*, 986 *m* cm^−1^; ^1^H NMR (400 MHz, CDCl_3_): δ = 8.01 (*brs*, 1H, COO-H), 5.64 (s, 1H, 12-H), 4.70 (d, *J* = 10.9 Hz, 1H_,_ 30-H_a_), 4.51 (dd, *J* = 11.5, 4.9 Hz, 1H, 3-H), 3.46 (d, *J* = 10.9 Hz, 1H, 30-H_b_), 2.85 (dt, *J* = 13.6, 3.5 Hz, 1H, 1-H_a_), 2.79–2.71 (m, 2H, 34-H), 2.58–2.49 (m, 2H, 35-H), 2.37 (s, 1H, 9-H), 2.36–2.25 (m, 1H, 18-H), 2.12–2.06 (m, 1H, 16-H_a_), 2.04 (s, 3H, 32-H), 1.87–1.75 (m, 1H, 15-H_a_), 1.74–1.36 (m, 9H, 2-H + 7-H_a_ + 6-H_a_ + 19-H_a_ + 6-H_b_ + 22-H_a_ + 7-H_b_ + 21-H_a_), 1.35 (s, 3H, 27-H), 1.33–1.14 (m, 4H, 21-H_b_ + 22-H_b_ + 19-H_b_ + 16-H_b_), 1.12 (s, 6H, 25-H + 26-H), 1.10– 0.98 (m, 2H, 1-H_b_ + 15-H_b_), 0.95 (s, 3H, 29-H), 0.87 (s, 9H, 28-H + 23-H + 24-H), 0.83–0.77 (m, 1H, 5-H) ppm; ^13^C NMR (101 MHz, CDCl_3_): δ = 202.2 (C-11), 174.8 (C-36), 172.9 (C-33), 171.8 (C-13), 171.1 (C-31), 128.1 (C-12), 80.7 (C-3), 67.7 (C-30), 61.9 (C-9), 55.2 (C-5), 46.7 (C-18), 45.5 (C-8), 43.4 (C-14), 39.2 (C-19), 39.0 (C-1), 38.3 (C-4), 37.4 (C-10), 36.1 (C-22), 34.9 (C-20), 32.7 (C-7), 32.3 (C-17), 31.3 (C-21), 29.5 (C-35), 28.9 (C-34), 28.9 (C-28), 28.2 (C-24), 28.2 (C-29), 26.6 (C-16), 26.6 (C-15), 23.7 (C-2), 23.3 (C-27), 21.4 (C-32), 19.0 (C-26), 17.5 (C-6), 16.8 (C-23), 16.7 (C-25) ppm; MS (ESI, MeOH/CHCl_3_, 4:1) *m/z* = 600.0 (96%, [M + H]^+^); analysis calcd for C_36_H_54_O_7_ (598.82): C 72.21, H 9.09; found: C 71.97, H 9.32.

### 4.17. (3β) 3-(Acetyloxy)olean-12-en-28-yl-4-{[5-(aminosulfonyl)-1,3,4-thiadiazol-2-yl]amino}-4-oxobutanoate

To a solution of 13 (0.250 g, 0.427 mmol) in dry THF (20 mL) at −15 °C, 4-methylmorpholine (0.1 mL, 0641 mmol) and ethyl chloroformate (0.05 mL, 0.513 mmol) were added, and the mixture was stirred for 10 min at this temperature. Then 5-amino-1,3,4-thiadiazole-2-sulfonamide 5 (0.092 g, 0.513 mmol) was added, and the mixture was heated under reflux for 4 h. The solvents were removed under diminished pressure, and the residue was subjected to chromatography (silica gel, (CHCl_3_/MeOH, 95:5) to yield 18 (0.166 g, 52%) as a colorless solid; m.p. 198–200 °C; R_F_ = 0.36 (silica gel, CHCl_3_/MeOH, 9:1); [α]_D_ = +19.2° (*c* = 0.122, CHCl_3_); UV-Vis (CHCl_3_): λ_max_ (log ε) = 262 nm (4.45); IR (ATR): ν = 3332*w*, 3234*w*, 2947*m*, 2864*m*, 1734*m*, 1704*s*, 1545*m*, 1536*m*, 1463*w*, 1432*w*, 1422*w*, 1371*s*, 1329*m*, 1245*s*, 1216*m*, 1173*s*, 1092*m*, 1027*m*, 1004*m*, 985*m*, 967*m*, 755*m*, 651*m*, 637*m*, 606 *s*, 504*m* cm^−1^; ^1^H NMR (500 MHz, DMSO-*d_6_*): δ = 13.07 (*brs*, 1H, CON-H), 8.30 (d, *J* = 6.2 Hz, 2H, SN-H), 5.15 (t, *J* = 3.7 Hz, 1H, 12-H), 4.38 (dd, *J* = 11.7, 4.5 Hz, 1H, 3-H), 3.96 (d, *J* = 10.9 Hz, 1H, 28-H_a_), 3.60 (d, *J* = 10.9 Hz, 1H, 28-H_b_), 2.89–2.76 (m, 2H, 34-H), 2.72–2.65 (m, 2H, 35-H), 2.02–1.97 (m, 1H, 18-H), 1.99 (s, 3H, 32-H), 1.86 (td, *J* = 13.9, 4.6 Hz, 1H, 16-H_a_), 1.82–1.75 (m, 2H, 11-H), 1.70 (dd, *J* = 13.4, 13.4 Hz, 1H, 19-H_a_), 1.62–1.13 (m, 11H, 15-H_a_ + 2-H + 1-H_a_ + 9-H + 6-H_a_ + 21-H_a_ + 22-H_a_, 6-H_b_ + 22-H_b_ + 7-H_b_), 1.11 (s, 3H, 27-H), 1.09–0.93 (m, 4H, 16-H_b_ + 7-H_b_ + 19-H_b_ + 1-H_b_), 0.88 (s, 3H, 25-H), 0.85 (d, 3H, 30-H), 0.84 (d, 3H, 29-H), 0.83 (s, 3H, 24-H), 0.83–0.80 (m, 7H, 26-H + 23-H + 5-H); ppm; ^13^C NMR (126 MHz, DMSO-*d_6_*): δ = 171.6 (C-33), 171.2 (C-36), 170.1 (C-31), 164.3 (C-38), 161.0 (C-37), 143.4 (C-13), 122.2 (C-12), 79.9 (C-3), 70.0 (C-28), 54.5 (C-5), 46.7 (C-9), 45.7 (C-19), 41.8 (C-18), 41.1 (C-14), 39.2 (C-8), 37.7 (C-1), 37.3 (C-4), 36.3 (C-10), 35.4 (C-17), 33.4 (C-7), 32.9 (C-30), 31.8 (C-21), 31.0 (C-22), 30.5 (C-20), 30.0 (C-34), 28.4 (C-35), 27.7 (C-24), 25.7 (C-27), 25.1 (C-15), 23.4 (C-29), 23.2 (C-2), 23.0 (C-11), 21.5 (C-16), 20.9 (C-32), 17.7 (C-6), 16.6 (C-26), 16.2 (C-23), 15.2 (C-25) ppm; MS (ESI, MeOH/CHCl_3_, 4:1): *m/z* = 770.1 (100%, [M + Na]^+^); analysis calcd for C_38_H_58_S_2_N_4_ (747.02): C 61.10, H 7.83, N 7.50; found: C 60.81, H 8.03, N 7.39.

### 4.18. (3β)-3-(Acetyloxy)urs-12-en-28-yl-4-{[5-(aminosulfonyl)-1,3,4-thiadiazol-2-yl]amino}4-oxobutanoate

Following the procedure given above for the synthesis of 18, from 14 (0.250 g, 0.427 mmol) 21 (0.217 g, 68%) was obtained as a colorless solid; m.p. 190–192 °C; R_F_ = 0.33 (silica gel, CHCl_3_/MeOH, 9:1); [α]_D_ = +27.4° (*c* = 0.129, CHCl_3_); UV-Vis (CHCl_3_): λ_max_ (log ε) = 263 nm (4.98); IR (ATR): ν = 3244*w*, 2948*m*, 2925*m*, 2871*m*, 1733*m*, 1705*s*, 1531*m*, 1457*w*, 1431*w*; 1414*w*, 1367*s*, 1245*s*, 1174*s*, 1094*m*, 1027*m*, 1006*m*, 985*m*, 967*m*, 655*m*, 603*s*, 508*m* cm^−1^; ^1^H NMR (500 MHz, DMSO-*d_6_*): δ = 13.07 (*brs*, 1H, CON-H), 8.30 (s, *J* = 2H, SN-H), 5.10 (t, *J* = 3.2 Hz, 1H, H-12), 4.38 (dd, *J* = 11.5, 4.7 Hz, 1H, H-3), 3.94 (d, *J* = 10.8 Hz, 1H, 28-H_a_), 3.54 (d, *J* = 10.9 Hz, 1H, 28-H_b_), 2.84–2.78 (m, 2H, 34-H), 2.71–2.65 (m, 2H, 35-H), 1.99 (s, 3H, 32-H), 1.92–1.80 (m, 3H, 16-H_a_ + 11-H), 1.65–1.53 (m, 3H, 15-H_a_ + 2-H_a_ + 1-H_a_), 1.53–1.42 (m, 5H, 2-H_a_ + 9-H + 7-H_a_ + 6-H_a_ + 22-H_a_), 1.40–1.06 (m, 7H, 18-H + 6-H_b_ + 21-H_a_ + 22-H_b_ + 7-H_b_ + 21-H_b_ + 16-H_b_), 1.04 (s, 3H, 27-H), 1.01–0.96 (m, 1H, 1-H_b_), 0.90 (s, 3H, 23-H), 0.87 (d, *J* = 6.7, 3H, 29-H), 0.86 (s, 3H, 26-H), 0.85–0.83 (m, 2H, 15-H_b_ + 1-H_b_), 0.83 (s, 6H, 24-H + 25-H), 0.83–0.80 (m, 1H, 20-H), 0.87 (d, *J* = 5.3 Hz, 3H, 30-H) ppm; ^13^C NMR (126 MHz, DMSO-*d_6_*): δ = 171.4 (C-33), 171.2 (C-36), 170.1 (C-31), 164.3 (C-38), 161.0 (C-37), 138.0 (C-13), 124.8 (C-12), 79.9 (C-3), 70.4 (C-28), 54.5 (C-5), 53.4 (C-18), 46.8 (C-9), 41.4 (C-14), 39.4 (C-8), 38.7 (C-20), 38.5 (C-19), 37.9 (C-1), 37.3 (C-4), 36.4 (C-17), 36.3 (C-10), 35.1 (C-2), 32.1 (C-7), 30.0 (C-34), 29.9 (C-21), 28.4 (C-35), 27.7 (C-24), 25.5 (C-15), 23.3 (C-2), 23.0 (C-27), 22.9 (C-11), 22.8 (C-16), 21.0 (C-29), 20.9 (C-32), 17.1 (C-6), 17.1 (C-30), 16.6 (C-23), 16.3 (C-26), 15.2 (C-25) ppm; MS (ESI, MeOH/CHCl_3_, 4:1): *m/z* = 770.1 (100%, [M + Na]^+^); analysis calcd for C_38_H_58_S_2_N_4_O_7_ (747.02): C 61.10, H 7.83, N 7.50; found: C 60.89, H 8.07, N 7.36.

### 4.19. (3β, 20β) 3-(Acetyloxy)-11-oxoolean-12-en-30-yl-4-{[5-(aminosulfonyl)-1,3,4-thiadiazol-2-yl] amino}-4-oxobutanoate

Following the procedure given above for the synthesis of 18, from 17 (0.250 g, 0.417 mmol) 20 (0.215 g, 68%) was obtained as a colorless solid; m.p. 179–181 °C; R_F_ = 0.41 (silica gel, CHCl_3_/MeOH, 9:1); [α]_D_ = +83.5° (*c* = 0.114, CHCl_3_); UV-Vis (CHCl_3_): λ_max_ (log ε) = 256 nm (4.25); IR (ATR): ν = 3252*w*, 2950*m*, 2872w, 1727*m*, 1709*m*, 1643*m*, 1528*m*, 1466*w*, 1455*w*, 1365*s*, 1323*m*, 1247*s*, 1215*m*, 1173*s*, 1088*w*, 1049*w*, 1028*m*, 1001*m*, 985*m*, 754*s*, 667*m*, 654*m*, 604*s*, 509*m* cm^−1^; ^1^H NMR (500 MHz, DMSO-*d_6_*): δ = 13.08 (*brs*, 1H, CON-H), 8.30 (s, *J* = 2H, SN-H), 5.48 (s, 1H, 12-H), 4.42 (dd, *J* = 11.8, 4.5 Hz, 1H, 3-H), 4.05 (d, *J* = 11.0 Hz, 1H, 30-H_a_), 3.92 (d, *J* = 11.0 Hz, 1H, 30-H_b_), 2.82 (dd, *J* = 7.5, 5.5 Hz 2H, 35-H), 2.71 (dd, *J* = 7.6, 5.5 Hz, 2H, 34-H), 2.61 (dt, *J* = 13.4, 3.6 Hz, 1H, 1-H_a_), 2.38 (s, 1H, 9-H), 2.16–2.04 (m, 2H, 18-H + 16-H_a_), 2.00 (s, 3H, 32-H), 1.79–1.35 (m, 7H, 16-H_b_ + 7-H_a_ + 19-H_a_ + 2-H_a_ + 6-H_a_ + 2-H_b_ + 6-H_b_). 1.34 (s, 3H, 27-H), 1.34–1.31 (m, 3H, 7-H_b_ + 21-H_b_ + 22-H_b_), 1.24–1.06 (m, 4H, 19-H_b_ + 22-H_b_ + 15-H_b_ + 1-H_b_), 1.06 (s, 1H, 25-H) 1.04 (s, 1H, 26-H)), 0.86– 0.98 (m, 2H, 15-H_b_ + 5-H), 0.85 (s, 3H, 29-H), 0.82 (s, 6H, 23-H + 24-H), 0.80 (s, 3H, 28-H) ppm; ^13^C NMR (126 MHz, DMSO-*d_6_*): δ = 198.9 (C-11), 171.8 (C-36), 171.1 (C-33), 170.1 (C-31), 170.0 (C-13), 127.4 (C-12), 79.7 (C-3), 67.0 (C-30), 60.8 (C-9), 53.7 (C-5), 46.0 (C-18), 44.9 (C-8), 43.0 (C-14), 39.7 (C-19), 37.8 (C-1), 37.5 (C-4), 36.5 (C-10), 35.4 (C-22), 34.0 (C-20), 31.9 (C-7), 31.8 (C-17), 29.9 (C-35), 29.3 (C-21), 28.3 (C-34), 28.1 (C-28), 27.7 (C-24), 27.2 (C-29), 25.9 (C-16), 25.9 (C-15), 23.2 (C-2), 23.1 (C-27), 20.9 (C-32), 18.3 (C-26), 16.9 (C-6), 16.6 (C-23), 16.1 (C-25) ppm; MS (ESI, MeOH/CHCl_3_, 4:1): *m/z* = 784.0 (100%, [M + Na]^+^; analysis calcd for C_38_H_56_S_2_N_4_O_8_ (761.00): C 59.97, H 7.42, N 7.36; found: C 59.71, H 7.71, N 7.09.

## Figures and Tables

**Table 1:** Inhibition percentage of conjugates (at 10 µM concentration) and of standard acetazolamide (**4**).

Compound	Inhibition [%]
**4**	89.9 ± 0.6
**6**	93.0 ± 0.1
**18**	49.4 ± 0.1
**19**	70.8 ± 0.2
**20**	96.8 ± 0.2

**Table 2:** K_i_ values (in μM) for conjugates **6**, **19,** and **20**.

Compound	K_i_ (in μM)
**6**	0.129 ± 0.02
**19**	0.91 ± 0.17
**20**	5.22 ± 0.57

**Table 3:** Cytotoxicity of acetazolamide (**4**) and conjugates **6**, **18-20** assessed from SRB-assays (EC_50_ values [µM] after 72 h of treatment). Human cancer cell lines: A375 (epithelial melanoma), HT29 (colorectal adenocarcinoma), MCF-7 (breast adenocarcinoma), A2780 (ovarian carcinoma); non-malignant: NIH 3T3 (fibroblasts); n.d. not determined; positive control: doxorubicin (**DX**).

	A375	HT29	MCF-7	A2780	NIH 3T3
**4**	>30	>30	>30	>30	>30
**6**	8.5 ± 0.7	10.2 ± 1.3	8.9 ± 0.7	9.3 ± 1.2	9.5 ± 1.0
**18**	10.1 ± 0.8	14.2 ± 1.4	10.6 ± 1.4	11.8 ± 1.4	14.0 ± 1.5
**19**	13.7 ± 1.1	15.0 ± 0.6	12.4 ± 0.8	12.5 ± 1.7	14.8 ± 1.5
**20**	9.2 ± 0.5	13.0 ± 1.3	10.5 ± 1.2	9.8 ± 0.8	11.9 ± 1.7
**DX**	n.d.	0.25 ± 0.02	0.1 ± 0.01	0.1 ± 0.01	0.01 ± 0.001

## Data Availability

The data presented in this study are available on request from the corresponding author.

## Sample Availability

Samples of the compounds are available from the authors.

## References

[1] Supuran C.T. (2008). Carbonic anhydrases—An overview. Curr. Pharm. Des..

[2] Supuran C.T. (2010). Carbonic anhydrase inhibitors. Bioorg. Med. Chem. Lett..

[3] Angeli A., Carta F., Nocentini A., Winum J.-Y., Zalubovskis R., Onnis V., Eldehna W.M., Capasso C., Carradori S., Donald W.A. (2021). Response to Perspectives on the Classical Enzyme Carbonic Anhydrase and the Search for Inhibitors. Biophys. J..

[4] Berrino E., Michelet B., Martin-Mingot A., Carta F., Supuran C.T., Thibaudeau S. (2021). Modulating the Efficacy of Carbonic Anhydrase Inhibitors through Fluorine Substitution. Angew. Chem. Int. Ed..

[5] Kumar S., Rulhania S., Jaswal S., Monga V. (2021). Recent advances in the medicinal chemistry of carbonic anhydrase inhibitors. Eur. J. Med. Chem..

[6] Supuran C.T. (2021). Emerging role of carbonic anhydrase inhibitors. Clin. Sci..

[7] Amedei A., Capasso C., Nannini G., Supuran C.T. (2021). Microbiota, bacterial carbonic anhydrases, and modulators of their activity: Links to human diseases?. Mediat. Inflamm..

[8] Campestre C., De Luca V., Carradori S., Grande R., Carginale V., Scaloni A., Supuran C.T., Capasso C. (2021). Carbonic Anhydrases: New Perspectives on Protein Functional Role and Inhibition in Helicobacter pylori. Front. Microbiol..

[9] Hines K.M., Chaudhari V., Edgeworth K.N., Owens T.G., Hanson M.R. (2021). Absence of carbonic anhydrase in chloroplasts affects C3 plant development but not photosynthesis. Proc. Natl. Acad. Sci. USA.

[10] Polishchuk O.V. (2021). Stress-Related Changes in the Expression and Activity of Plant Carbonic Anhydrases. Planta.

[11] Rudenko N.N., Ignatova L.K., Nadeeva-Zhurikova E.M., Fedorchuk T.P., Ivanov B.N., Borisova-Mubarakshina M.M. (2021). Advances in understanding the physiological role and locations of carbonic anhydrases in C3 plant cells. Protoplasma.

[12] Weerasooriya H.N., DiMario R.J., Rosati V.C., Rai A.K., LaPlace L.M., Filloon V.D., Longstreth D.J., Moroney J.V. (2022). Arabidopsis plastid carbonic anhydrase βCA5 is important for normal plant growth. Plant Physiol..

[13] Buabeng E.R., Henary M. (2021). Developments of small molecules as inhibitors for carbonic anhydrase isoforms. Bioorg. Med. Chem..

[14] Elimam D.M., Elgazar A.A., Bonardi A., Abdelfadil M., Nocentini A., El-Domany R.A., Abdel-Aziz H.A., Badria F.A., Supuran C.T., Eldehna W.M. (2021). Natural inspired piperine-based sulfonamides and carboxylic acids as carbonic anhydrase inhibitors: Design, synthesis and biological evaluation. Eur. J. Med. Chem..

[15] Kalinin S., Malkova A., Sharonova T., Sharoyko V., Bunev A., Supuran C.T., Krasavin M. (2021). Carbonic Anhydrase IX Inhibitors as Candidates for Combination Therapy of Solid Tumors. Int. J. Mol. Sci..

[16] Nerella S.G., Singh P., Arifuddin M., Supuran C.T. (2022). Anticancer carbonic anhydrase inhibitors: A patent and literature update 2018–2022. Expert Opin. Ther. Pat..

[17] Shaldam M., Eldehna W.M., Nocentini A., Elsayed Z.M., Ibrahim T.M., Salem R., El-Domany R.A., Capasso C., Abdel-Aziz H.A., Supuran C.T. (2021). Development of novel benzofuran-based SLC-0111 analogs as selective cancer-associated carbonic anhydrase isoform IX inhibitors. Eur. J. Med. Chem..

[18] Testa C., Papini A.M., Zeidler R., Vullo D., Carta F., Supuran C.T., Rovero P. (2022). First studies on tumor associated carbonic anhydrases IX and XII monoclonal antibodies conjugated to small molecule inhibitors. J. Enzym. Inhib. Med. Chem..

[19] Mincione F., Nocentini A., Supuran C.T. (2021). Advances in the discovery of novel agents for the treatment of glaucoma. Expert Opin. Drug Discov..

[20] Ozsoy H.Z. (2021). Anticonvulsant Effects of Carbonic Anhydrase Inhibitors: The Enigmatic Link Between Carbonic Anhydrases and Electrical Activity of the Brain. Neurochem. Res..

[21] Supuran C.T. (2021). Novel carbonic anhydrase inhibitors. Future Med. Chem..

[22] Akgul O., Lucarini E., Mannelli L.D.C., Ghelardini C., D’Ambrosio K., Buonanno M., Monti S.M., De Simone G., Angeli A., Supuran C.T. (2022). Sultam based Carbonic Anhydrase VII inhibitors for the management of neuropathic pain. Eur. J. Med. Chem..

[23] Kumar A., Agarwal P., Rathi E., Kini S.G. (2022). Computer-aided identification of human carbonic anhydrase isoenzyme VII inhibitors as potential antiepileptic agents. J. Biomol. Struct. Dyn..

[24] Magheru C., Magheru S., Coltau M., Hoza A., Moldovan C., Sachelarie L., Gradinaru I., Hurjui L.L., Marc F., Farcas D.M. (2022). Antiepileptic Drugs and Their Dual Mechanism of Action on Carbonic Anhydrase. J. Clin. Med..

[25] Ozaslan M.S., Saglamtas R., Demir Y., Genc Y., Saracoglu I., Guelcin I. (2022). Isolation of Some Phenolic Compounds from Plantago subulata L. and Determination of Their Antidiabetic, Anticholinesterase, Antiepileptic and Antioxidant Activity. Chem. Biodivers..

[26] Shukralla A.A., Dolan E., Delanty N. (2022). Acetazolamide: Old drug, new evidence?. Epilepsia Open.

[27] Das Mahapatra A., Queen A., Yousuf M., Khan P., Hussain A., Rehman T.M., Alajmi M.F., Datta B., Hassan I.M. (2022). Design and development of 5-(4H)-oxazolones as potential inhibitors of human carbonic anhydrase VA: Towards therapeutic management of diabetes and obesity. J. Biomol. Struct. Dyn..

[28] Mori M., Supuran C.T. (2022). Acipimox inhibits human carbonic anhydrases. J. Enzym. Inhib. Med. Chem..

[29] Supuran C.T. (2022). Anti-obesity carbonic anhydrase inhibitors: Challenges and opportunities. J. Enzym. Inhib. Med. Chem..

[30] Lemon N., Canepa E., Ilies M.A., Fossati S. (2021). Carbonic anhydrases as potential targets against neurovascular unit dysfunction in Alzheimer’s disease and stroke. Front. Aging Neurosci..

[31] Poggetti V., Salerno S., Baglini E., Barresi E., Da Settimo F., Taliani S. (2022). Carbonic Anhydrase Activators for Neurodegeneration: An Overview. Molecules.

[32] Bonardi A., Micheli L., Di Cesare Mannelli L., Ghelardini C., Gratteri P., Nocentini A., Supuran C.T. (2022). Development of Hydrogen Sulfide-Releasing Carbonic Anhydrases IX- and XII-Selective Inhibitors with Enhanced Antihyperalgesic Action in a Rat Model of Arthritis. J. Med. Chem..

[33] Bulli I., Dettori I., Coppi E., Cherchi F., Venturini M., Mannelli L.D.C., Ghelardini C., Nocentini A., Supuran C.T., Pugliese A.M. (2021). Role of carbonic anhydrase in cerebral ischemia and carbonic anhydrase inhibitors as putative protective agents. Int. J. Mol. Sci..

[34] Dettori I., Fusco I., Bulli I., Gaviano L., Coppi E., Cherchi F., Venturini M., Di Cesare Mannelli L., Ghelardini C., Nocentini A. (2021). Protective effects of carbonic anhydrase inhibition in brain ischaemia in vitro and in vivo models. J. Enzym. Inhib. Med. Chem..

[35] D’Agostino I., Mathew G.E., Angelini P., Venanzoni R., Angeles Flores G., Angeli A., Carradori S., Marinacci B., Menghini L., Abdelgawad M.A. (2022). Biological investigation of N-methyl thiosemicarbazones as antimicrobial agents and bacterial carbonic anhydrases inhibitors. J. Enzym. Inhib. Med. Chem..

[36] De Luca V., Carginale V., Supuran C.T., Capasso C. (2022). The gram-negative bacterium Escherichia coli as a model for testing the effect of carbonic anhydrase inhibition on bacterial growth. J. Enzym. Inhib. Med. Chem..

[37] Giovannuzzi S., Hewitt C.S., Nocentini A., Capasso C., Costantino G., Flaherty D.P., Supuran C.T. (2022). Inhibition studies of bacterial α-carbonic anhydrases with phenols. J. Enzym. Inhib. Med. Chem..

[38] Giovannuzzi S., Hewitt C.S., Nocentini A., Capasso C., Flaherty D.P., Supuran C.T. (2022). Coumarins effectively inhibit bacterial α-carbonic anhydrases. J. Enzym. Inhib. Med. Chem..

[39] Gueller P., Atmaca U., Gueller U., Calisir U., Dursun F. (2021). Antibacterial properties and carbonic anhydrase inhibition profiles of azido sulfonyl carbamate derivatives. Future Med. Chem..

[40] Hewitt C.S., Abutaleb N.S., Elhassanny A.E.M., Nocentini A., Cao X., Amos D.P., Youse M.S., Holly K.J., Marapaka A., An W. (2021). Structure-Activity Relationship Studies of Acetazolamide-Based Carbonic Anhydrase Inhibitors with Activity against Neisseria gonorrhoeae. ACS Infect. Dis..

[41] Nocentini A., Hewitt C.S., Mastrolorenzo M.D., Flaherty D.P., Supuran C.T. (2021). Anion inhibition studies of the α-carbonic anhydrases from Neisseria gonorrhoeae. J. Enzym. Inhib. Med. Chem..

[42] Flaherty D.P., Seleem M.N., Supuran C.T. (2021). Bacterial carbonic anhydrases: Underexploited antibacterial therapeutic targets. Future Med. Chem..

[43] Artasensi A., Angeli A., Lammi C., Bollati C., Gervasoni S., Baron G., Matucci R., Supuran C.T., Vistoli G., Fumagalli L. (2022). Discovery of a Potent and Highly Selective Dipeptidyl Peptidase IV and Carbonic Anhydrase Inhibitor as “Antidiabesity” Agents Based on Repurposing and Morphing of WB-4101. J. Med. Chem..

[44] Carradori S., Fantacuzzi M., Ammazzalorso A., Angeli A., De Filippis B., Galati S., Petzer A., Petzer J.P., Poli G., Tuccinardi T. (2022). Resveratrol Analogues as Dual Inhibitors of Monoamine Oxidase B and Carbonic Anhydrase VII: A New Multi-Target Combination for Neurodegenerative Diseases?. Molecules.

[45] Kalayci M., Tuerkes C., Arslan M., Demir Y., Beydemir S. (2021). Novel benzoic acid derivatives: Synthesis and biological evaluation as multitarget acetylcholinesterase and carbonic anhydrase inhibitors. Arch. Pharm..

[46] Lenci E., Angeli A., Calugi L., Innocenti R., Carta F., Supuran C.T., Trabocchi A. (2021). Multitargeting application of proline-derived peptidomimetics addressing cancer-related human matrix metalloproteinase 9 and carbonic anhydrase II. Eur. J. Med. Chem..

[47] Supuran C.T. (2021). Multitargeting approaches involving carbonic anhydrase inhibitors: Hybrid drugs against a variety of disorders. J. Enzym. Inhib. Med. Chem..

[48] Elbadawi M.M., Eldehna W.M., Nocentini A., Abo-Ashour M.F., Elkaeed E.B., Abdelgawad M.A., Alharbi K.S., Abdel-Aziz H.A., Supuran C.T., Gratteri P. (2021). Identification of N-phenyl-2-(phenylsulfonyl)acetamides/propanamides as new SLC-0111 analogues: Synthesis and evaluation of the carbonic anhydrase inhibitory activities. Eur. J. Med. Chem..

[49] Huo Z., Bilang R., Supuran C.T., von der Weid N., Bruder E., Holland-Cunz S., Martin I., Muraro M.G., Gros S.J. (2022). Perfusion-Based Bioreactor Culture and Isothermal Microcalorimetry for Preclinical Drug Testing with the Carbonic Anhydrase Inhibitor SLC-0111 in Patient-Derived Neuroblastoma. Int. J. Mol. Sci..

[50] Kumar A., Siwach K., Supuran C.T., Sharma P.K. (2022). A decade of tail-approach based design of selective as well as potent tumor associated carbonic anhydrase inhibitors. Bioorg. Chem..

[51] Mboge M.Y., Combs J., Singh S., Andring J., Wolff A., Tu C., Zhang Z., McKenna R., Frost S.C. (2021). Inhibition of Carbonic Anhydrase Using SLC-149: Support for a Noncatalytic Function of CAIX in Breast Cancer. J. Med. Chem..

[52] Mussi S., Rezzola S., Chiodelli P., Nocentini A., Supuran C.T., Ronca R. (2022). Antiproliferative effects of sulphonamide carbonic anhydrase inhibitors C18, SLC-0111 and acetazolamide on bladder, glioblastoma and pancreatic cancer cell lines. J. Enzym. Inhib. Med. Chem..

[53] Supuran C.T. (2021). Carbonic anhydrase inhibitors: An update on experimental agents for the treatment and imaging of hypoxic tumors. Expert Opin. Invest. Drugs.

[54] McDonald P.C., Chia S., Bedard P.L., Chu Q., Lyle M., Tang L., Singh M., Zhang Z., Supuran C.T., Renouf D.J. (2020). A Phase 1 Study of SLC-0111, a Novel Inhibitor of Carbonic Anhydrase IX, in Patients With Advanced Solid Tumors. Am. J. Clin. Oncol..

[55] Amiri A., Le P.U., Moquin A., Machkalyan G., Petrecca K., Gillard J.W., Yoganathan N., Maysinger D. (2016). Inhibition of carbonic anhydrase IX in glioblastoma multiforme. Eur. J. Pharm. Biopharm..

[56] Haapasalo J., Hilvo M., Nordfors K., Haapasalo H., Parkkila S., Hyrskyluoto A., Rantala I., Waheed A., Sly W.S., Pastorekova S. (2008). Identification of an alternatively spliced isoform of carbonic anhydrase XII in diffusely infiltrating astrocytic gliomas. Neuro Oncol..

[57] Haapasalo J., Nordfors K., Jarvela S., Bragge H., Rantala I., Parkkila A.-K., Haapasalo H., Parkkila S. (2007). Carbonic anhydrase II in the endothelium of glial tumors: A potential target for therapy. Neuro Oncol..

[58] Ihnatko R., Kubes M., Takacova M., Sedlakova O., Sedlak J., Pastorek J., Kopacek J., Pastorekova S. (2006). Extracellular acidosis elevates carbonic anhydrase IX in human glioblastoma cells via transcriptional modulation that does not depend on hypoxia. Int. J. Oncol..

[59] Ivanov S., Liao S.-Y., Ivanova A., Danilkovitch-Miagkova A., Tarasova N., Weirich G., Merrill M.J., Proescholdt M.A., Oldfield E.H., Lee J. (2001). Expression of hypoxia-inducible cell-surface transmembrane carbonic anhydrases in human cancer. Am. J. Pathol..

[60] Korkolopoulou P., Perdiki M., Thymara I., Boviatsis E., Agrogiannis G., Kotsiakis X., Angelidakis D., Rologis D., Diamantopoulou K., Thomas-Tsagli E. (2007). Expression of hypoxia-related tissue factors in astrocytic gliomas. A multivariate survival study with emphasis upon carbonic anhydrase IX. Hum. Pathol..

[61] Nordfors K., Haapasalo J., Korja M., Niemela A., Laine J., Parkkila A.-K., Pastorekova S., Pastorek J., Waheed A., Sly W.S. (2010). The tumour-associated carbonic anhydrases CA II, CA IX and CA XII in a group of medulloblastomas and supratentorial primitive neuroectodermal tumours: An association of CA IX with poor prognosis. BMC Cancer.

[62] Proescholdt M.A., Mayer C., Kubitza M., Schubert T., Liao S.-Y., Stanbridge E.J., Ivanov S., Oldfield E.H., Brawanski A., Merrill M.J. (2005). Expression of hypoxia-inducible carbonic anhydrases in brain tumors. Neuro Oncol..

[63] Proescholdt M.A., Merrill M.J., Stoerr E.-M., Lohmeier A., Pohl F., Brawanski A. (2012). Function of carbonic anhydrase IX in glioblastoma multiforme. Neuro Oncol..

[64] Said H.M., Supuran C.T., Hageman C., Staab A., Polat B., Katzer A., Scozzafava A., Anacker J., Flentje M., Vordermark D. (2010). Modulation of carbonic anhydrase 9 (CA9) in human brain cancer. Curr. Pharm. Des..

[65] Boyd N.H., Walker K., Fried J., Hackney J.R., McDonald P.C., Benavides G.A., Spina R., Audia A., Scott S.E., Landis C.J. (2017). Addition of carbonic anhydrase 9 inhibitor SLC-0111 to temozolomide treatment delays glioblastoma growth in vivo. JCI Insight.

[66] Mujumdar P., Kopecka J., Bua S., Supuran C.T., Riganti C., Poulsen S.-A. (2019). Carbonic Anhydrase XII Inhibitors Overcome Temozolomide Resistance in Glioblastoma. J. Med. Chem..

[67] Salaroglio I.C., Mujumdar P., Annovazzi L., Kopecka J., Mellai M., Schiffer D., Poulsen S.-A., Riganti C. (2018). Carbonic anhydrase XII inhibitors overcome P-glycoprotein-mediated resistance to temozolomide in glioblastoma. Mol. Cancer Ther..

[68] Tann A.C., Ashley D.M., López G.Y., Malinzak M., Friedman H.S. (2020). Khasraw, M. Mangement of glioblastoma: State of the art andfuture directions. S. CA Cancer J. Clin..

[69] Zhao K., Schaefer A., Zhang Z., Elsaesser K., Culmsee C., Zhong L., Pagenstecher A., Nimsky C., Bartsch J.W. (2022). Inhibition of Carbonic Anhydrase 2 Overcomes Temozolomide Resistance in Glioblastoma Cells. Int. J. Mol. Sci..

[70] Hannen R., Selmansberger M., Hauswald M., Pagenstecher A., Nist A., Stiewe T., Acker T., Carl B., Nimsky C., Bartsch J.W. (2019). Comparative transcriptomic analysis of temozolomide resistant primary GBM stem-like cells and recurrent GBM identifies upo-regulation of carbonic anhydrase CA2 gene as resistance factor. Cancers.

[71] Schwarz S., Sommerwerk S., Lucas S.D., Heller L., Csuk R. (2014). Sulfamates of methyl triterpenoates are effective and competitive inhibitors of carbonic anhydrase II. Eur. J. Med. Chem..

[72] Rehman N.U., Halim S.A., Khan A., Khan M., Al-Hatmi S., Al-Harrasi A. (2022). Commikuanoids A-C: New cycloartane triterpenoids with exploration of carbonic anhydrase-II inhibition from the resins of Commiphora kua by in vitro and in silico molecular docking. Fitoterapia.

[73] Silva V.A.O., Rosa M.N., Miranda-Goncalves V., Costa A.M., Tansini A., Evangelista A.F., Martinho O., Carloni A.C., Jones C., Lima J.P. (2019). Euphol, a tetracyclic triterpene, from Euphorbia tirucalli induces autophagy and sensitizes temozolomide cytotoxicity on glioblastoma cells. Invest. New Drugs.

[74] Avula S.K., Rehman N.U., Khan M., Halim S.A., Khan A., Rafiq K., Csuk R., Das B., Al-Harrasi A. (2021). New synthetic 1H-1,2,3-triazole derivatives of 3-O-acetyl-β-boswellic acid and 3-O-acetyl-11-keto-β-boswellic acid from Boswellia sacra inhibit carbonic anhydrase II in vitro. Med. Chem. Res..

[75] Bulbul M., Saracoglu N., Irfan Kufrevioglu O., Ciftci M. (2002). Bile acid derivatives of 5-amino-1,3,4-thiadiazole-2-sulfonamide as new carbonic anhydrase inhibitors: Synthesis and investigation of inhibition effects. Bioorg. Med. Chem..

[76] Chu X., Battle C.H., Zhang N., Aryal G.H., Mottamal M., Jayawickramarajah J. (2015). Bile Acid Conjugated DNA Chimera that Conditionally Inhibits Carbonic Anhydrase-II in the Presence of MicroRNA-21. Bioconjugate Chem..

[77] Scozzafava A., Supuran C.T. (2002). Carbonic anhydrase inhibitors. Preparation of potent sulfonamides inhibitors incorporating bile acid tails. Bioorg. Med. Chem. Lett..

[78] Trifunovic J., Borcic V., Mikov M. (2017). Bile acids and their oxo derivatives: Potential inhibitors of carbonic anhydrase I and II, androgen receptor antagonists and CYP3A4 substrates. Biomed. Chromatogr..

[79] Kalyanavenkatapaman S., Nanjen P., Banerji A., Nair B.C., Kumar G.B. (2016). Discovery of arjunolic acid as a novel non-zinc binding carbonic anhydrase II inhibitor. Bioorg. Chem..

[80] Pastorekova S., Gillies R.J.M. (2019). The role of carbonic anhydrase IX in cancer development: Links to hypxia, acidosis and beyond. Cancer Metastasis Rev..

[81] Vanchanagiri K., Emmerich D., Brusche M., Bache M., Seifert F., Csuk R., Vordermark D., Paschke R. (2018). Synthesis and biological investigation of new carbonic anhydrase IX (CAIX) inhibitors. Chem. Biol. Interact..

[82] Annan D.A., Maishi N., Soga T., Bawood R., Li C., Kikuchi H.K., Hajo T., Morimoto M., Kitamura T., Alam M.T. (2019). Carbonic anhydrase 2 (CA II) supports tumor blood endothelial cell survival under lactic acidosis in the tumor microenvironment. Cell Commun. Signal..

[83] Bekku S., Mochizuki H., Takayama E., Shinomiya N., Fukamachi H., Ichinose M., Tadakuma T., Yamamoto T. (1998). Carbonic anhydrase I and II as a differntiation marker of human and rat colonic enterocytes. Res. Exp. Med..

[84] Noor S.I., Jamali S., Ames S., Langer S., Deitmer J.W., Becker H.M. (2018). A surface proton antenna in carbonic anhydrase II supports lactate transport in cancer cells. eLife.

[85] Thibeault D., Gauthier C., Legault J., Bouchard J., Dufour P., Pichette A. (2007). Synthesis and structure–activity relationship study of cytotoxic germanicane- and lupane-type 3β-O-monodesmosidic saponins starting from betulin. Bioorganic Med. Chem..

[86] Flekhter O.B., Karachurina L.T., Poroikov V.V., Nigmatullina L.P., Baltina L.A., Zarudii F.S., Davydova V.A., Spirikhin L.V., Baikova I.P., Galin F.Z. (2000). The synthesis and hepatoprotective activity of esters of the lupane group triterpenoids. Russ. J. Bioorg. Chem..

[87] Flekhter O.B., Medvedeva N.I., Karachurina L.T., Baltina L.A., Galin F.Z., Zarudii F.S., Tolstikov G.A. (2005). Synthesis and Pharmacological Activity of Betulin, Betulinic Acid, and Allobetulin Esters. Pharm. Chem. J..

[88] Kazakova O.B., Smirnova I.E., Baltina L.A., Boreko E.I., Savinova O.V., Pokrovskii A.G. (2018). Antiviral Activity of Acyl Derivatives of Betulin and Betulinic and Dihydroquinopimaric Acids. Russ. J. Bioorg. Chem..

[89] Komissarova N.G., Dubovitskii S.N., Orlov A.V., Shitikova O.V. (2019). New Conjugates of Betulin with 2-Aminoethanesulfonic Acid. Chem. Nat. Compd..

[90] Pokrovskii A.G., Plyasunvoa O.A., Il’icheva T.N., Borisova O.A., Fedyuk N.V., Petrenko N.I., Petukhova V.Z., Shul’ts E.E., Tolstikov G.A. (2001). Synthesis of derivatives of plant triterpenes and study of their antiviral and immunostimulating activity. Khim. Interes. Ustoich. Razvit..

[91] Shintyapina A.B., Shults E.E., Petrenko N.I., Uzenkova N.V., Tolstikov G.A., Pronkina N.V., Kozhevnikov V.S., Pokrovsky A.G. (2007). Effect of nitrogen-containing derivatives of the plant triterpenes betulin and glycyrrhetic acid on the growth of MT-4, MOLT-4, CEM, and Hep G2 tumor cells. Russ. J. Bioorg. Chem..

[92] Roblin R.O., Clapp J.W. (1950). The preparation of heterocyclic sulfonamides. J. Am. Chem. Soc..

[93] Kazakova O.B., Giniyatullina G.V., Tolstikov G.A., Baikova I.P., Zaprutko L., Apryshko G.N. (2011). Synthesis and antitumor activity of aminopropoxy derivatives of betulin, erythrodiol, and uvaol. Russ. J. Bioorg. Chem..

[94] Zimmermann J. (1932). A monostearic ester of a triterpenediol from the fruits of Erythroxylon novogranatense. Recl. Trav. Chim. Pays-Bas Belg..

[95] Rollinger J.M., Kratschmar D.V., Schuster D., Pfisterer P.H., Gumy C., Aubry E.M., Brandstoetter S., Stuppner H., Wolber G., Odermatt A. (2010). 11β-Hydroxysteroid dehydrogenase 1 inhibiting constituents from Eriobotrya japonica revealed by bioactivity-guided isolation and computational approaches. Bioorg. Med. Chem..

[96] Agarwal K.P., Roy A.C., Dhar M.L. (1963). Triterpenes from the bark of Myrica esculenta. Indian J. Chem..

[97] Garcia-Granados A., Lopez P.E., Melguizo E., Parra A., Simeo Y. (2004). Partial synthesis of C-ring derivatives from oleanolic and maslinic acids. Formation of several triene systems by chemical and photochemical isomerization processes. Tetrahedron.

[98] El-Seedi H.R. (2005). Antimicrobial triterpenes from Poulsenia armata Mif. Standl. Nat. Prod. Res..

[99] Kim M.-R., Lee H.-H., Hahm K.-S., Moon Y.-H., Woo E.-R. (2004). Pentacyclic triterpenoids and their cytotoxicity from the stem bark of Styrax japonica S. et Z. Arch. Pharmacal Res..

[100] Alves H.M., Arndt V.H., Ollis W.D., Eyton W.B., Gottlieb O.R., Magalhaes M.T. (1965). Chemistry of Brazilian leguminosae. VIII. β-Amyrin constituents of Machaerium incorruptibile. An. Acad. Bras. Cienc..

[101] Sengupta P., Dey A.K., Mukherjee J., Ghosh S., Das K.G. (1969). Terpenoids and related compounds. XVI. Terpenoids of the bark of Rhododendron falconeri. J. Indian Chem. Soc..

[102] Chen D., Xu F., Zhang P., Deng J., Sun H., Wen X., Liu J. (2017). Practical Synthesis of α-Amyrin, β-Amyrin, and Lupeol: The Potential Natural Inhibitors of Human Oxidosqualene Cyclase. Arch. Pharm..

[103] Beseda I., Czollner L., Shah P.S., Khunt R., Gaware R., Kosma P., Stanetty C., del Ruiz-Ruiz M.C., Amer H., Mereiter K. (2010). Synthesis of glycyrrhetinic acid derivatives for the treatment of metabolic diseases. Bioorg. Med. Chem..

